# Electrical response of fungi to changing moisture content

**DOI:** 10.1186/s40694-023-00155-0

**Published:** 2023-04-03

**Authors:** Neil Phillips, Antoni Gandia, Andrew Adamatzky

**Affiliations:** 1 Unconventional Computing Laboratory, Faculty of Environment and Technology, University of the West of England, Bristol, UK; 2 Institute for Plant Molecular and Cell Biology, CSIC-UPV, Valencia, Spain

**Keywords:** Mycelium network, Fungal materials, Environmental sensors, Oscillations, Biosensor

## Abstract

Mycelium-bound composites are potential alternatives to conventional materials for a variety of applications, including thermal and acoustic building panels and product packaging. If the reactions of live mycelium to environmental conditions and stimuli are taken into account, it is possible to create functioning fungal materials. Thus, active building components, sensory wearables, etc. might be created. This research describes the electrical sensitivity of fungus to changes in the moisture content of a mycelium-bound composite. Trains of electrical spikes initiate spontaneously in fresh mycelium-bound composites with a moisture content between $\sim$ 95% and $\sim$ 65%, and between $\sim$ 15% and $\sim$ 5% when partially dried. When the surfaces of mycelium-bound composites were partially or totally encased with an impermeable layer, increased electrical activity was observed. In fresh mycelium-bound composites, electrical spikes were seen both spontaneously and when induced by water droplets on the surface. Also explored is the link between electrical activity and electrode depth. Future designs of smart buildings, wearables, fungi-based sensors, and unconventional computer systems may benefit from fungi configurations and biofabrication flexibility.

## Introduction

Mycelium-bound composites—masses of organic substrates colonised by fungi—are considered environmentally friendly biodegradable biomaterials [1–4]. These fungal-based materials can be used in thermal insulation wall cladding [5–10], acoustic insulation panels [11–13], packaging materials [14–16], wearables [1, 17–22], art [23, 24], and interior design [24, 25]. Fungi are frugal colonisers and are found in most habitats on Earth wherever there is a minimal moisture availability. Remarkably, they can thrive in deserted areas thanks to symbiotic relationships with photosynthetic organisms like algae and plants. To this day, most fungal materials are finished and served in a dehydrated form that stops all biological activity throughout the substrate and avoids the eventual regrowth, sporulation, or further decay of the pieces and derived bioburden [12, 26]. Furthermore, downstream preservation techniques such as paint coatings and plasticisation are commonly used to extend the lifespan of these biomaterials to properly fit their functional and commercial purpose as decorative or architectural elements.

Contrary to common practice, in [27] we proposed to develop a structural substrate by using non-dehydrated living fungal mycelium, functionalise the substrate with nanoparticles and polymers to make mycelium-based electronics [28–31], and implement sensorial fusion and decision making in the mycelium networks [32]. Following that vision, the structural substrate—the living mycelium-bound composites—will be used to grow monolithic buildings from the functionalised fungal substrate [33]. Buildings grown with mycelium-bound composites could provide intelligent sensory capability if some parts of the mycelium remain alive, therefore securing a minimal viable moisture content will be crucial to keep the sensorial network of the fungus electrically active. In this case the fungal materials might be able to detect structural loads (dead loads such as the weight of the structure, live loads such as vehicle traffic, building contents, etc, and environmental loads such as wind, snow, etc) [34], illumination [31], temperature, and air pollution.

As part of our research into the sensing characteristics of fungus, we demonstrate in this paper how mycelium-bound composites respond to variations in moisture content by modifying their electrical activity. We chose electrical activity as an indicator of fungal response because fungi have been shown to respond to chemical and physical stimuli by changing patterns of electrical activity [35–37] and electrical properties [31].

## Methods and materials

### Moisture content mapped to electrical conductivity

Blocks of spawn substrate were bought from commercial suppliers. They were made of rye seeds and millet grain and were well colonised with two types of fungi: *Hericium erinaceus* (supplied by Urban Farm It Ltd, UK, product code M9514) and *Pleurotus ostreatus* (supplied by Mycelia BVBA, BE, product code M2125).

**Fig. 1:**
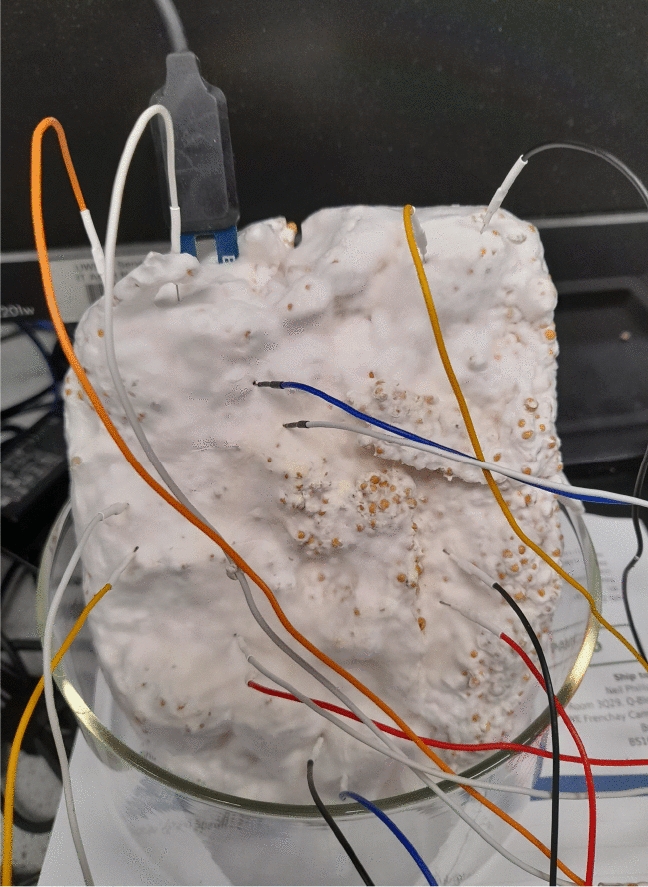
HOBO EC-5 moisture probe and sub-dermal needle electrodes inserted into unwrapped blocks of substrate colonised with *Pleurotus ostreatus*

A moisture probe (HOBO EC-5, Tempcon Instrumentation Ltd, UK) was inserted into blocks of colonised substrate and connected to a data logger (HOBO H21 USB Micro Station, Tempcon Instrumentation Ltd, UK). Every ten seconds, the electrical conductivity between the probe’s two electrodes was measured and saved (using HOBOware Pro Software from Tempcon Instrumentation Ltd, UK) on a Windows 10 computer for later analysis, see Fig. 1.

The following steps were taken to calibrate the moisture probe: (1) The sample is weighed, and the electrical conductivity between the probe electrodes is measured. (2) The sample is dried in an oven at 80 $^{\circ }$C for $\sim$ 48 h. The sample is weighed again, and then the electrical conductivity between the probe electrodes is measured while the sample is ‘bone dry.’ (4) The difference in weight and electrical conductivity between the two situations is calculated.

### Electrical activity mapped to moisture content

A freshly unwrapped ($\sim$ 500 g) block of substrate (rye seeds and millet grain) that had been colonised by *Pleurotus ostreatus* was left to slowly dehydrate at room temperature (18 $^{\circ }$C to 22 $^{\circ }$C) and ambient humidity ($\sim$ 30%). A calibrated HOBO EC-5 moisture probe was used to monitore the substrate’s moisture content (as previously described). A high-resolution data logger with a 24-bit A/D converter (ADC-24, PICO Technology, UK) and software (PicoLog 6, PICO Technology, UK) with a selectable sample rate and pairs of stainless steel sub-dermal needle electrodes were used to record electrical activity (Spes Medica S.r.l., IT). The sampling period was one second, which was used to record electrical activity. During the recording, the logger took as many measurements as it could (usually up to 600 per second) and saved the average value. The voltage range for acquisition was set to ±39 mV. Each pair of electrodes, called a channel (Ch), reported a difference in the electrical potential between them. The electrodes were pierced through the mycelium on the substrate’s surface. The distance between electrodes was 1 cm to 2 cm, see Fig. 1.

In a different experiment, a fresh block ($\sim$ 500 g) of substrate (rye grain seeds and millet grain) that had been colonised by *Pleurotus ostreatus* was left partly wrapped inside the plastic bag it was supplied in. The top of the bag was left open so that the substrate could slowly lose its moisture content. Subdermal needle electrodes with a length of 18 mm length were pushed through the plastic bag and $\sim$ 15 mm into the body of the substrate.

### Rate of water loss from substrate colonised with fungi

**Fig. 2:**
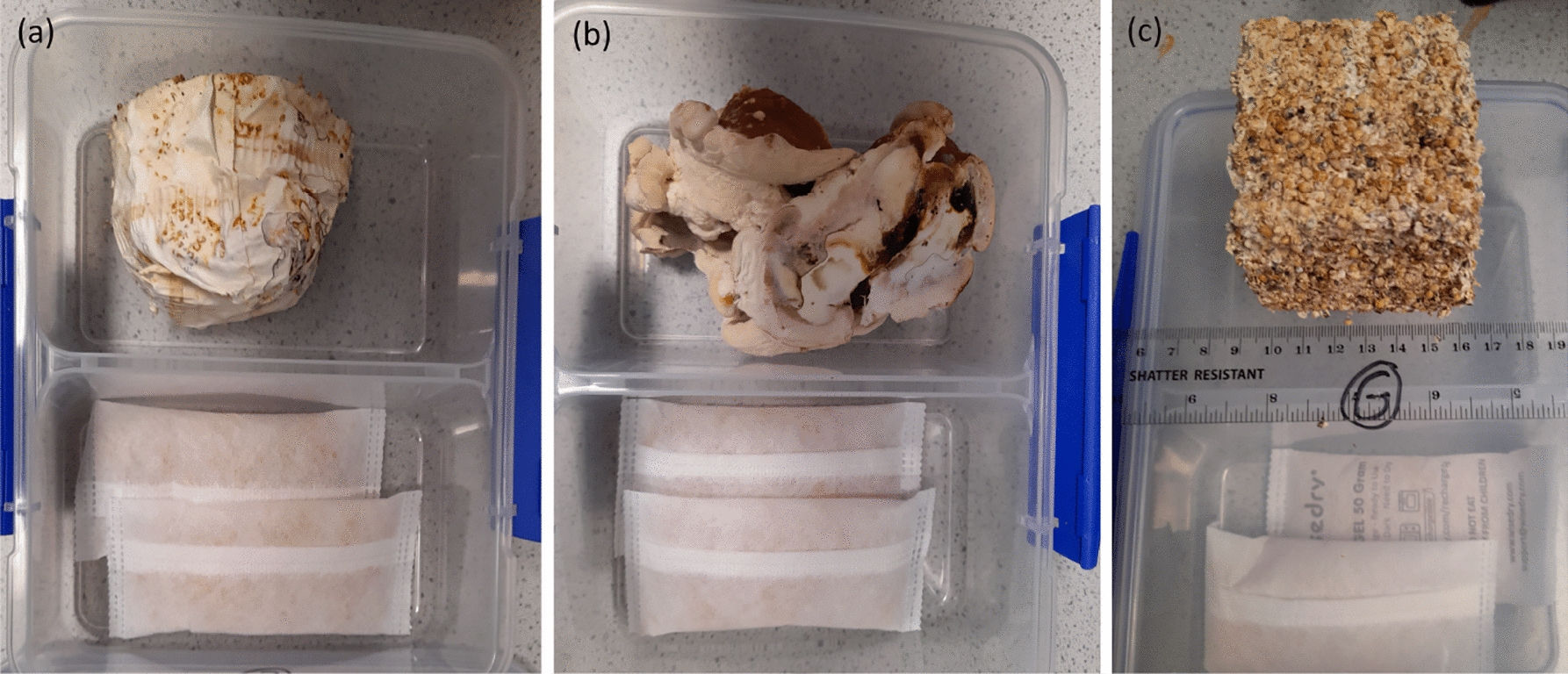
Drying containers with (**a**) block of colonised substrate with mycelium surface (**b**) fruiting bodies (**c**) block of colonised substrate with bare surface

5 L bag of substrate (rye and millet grain seed) well colonised with *Ganoderma lucidum* (manufactured by Mycelia BVBA, product code M9726, https://mycelia.be/shop/m9726-ganoderma-resinaceum/) was divided into the following ten samples (plus waste):

- 4 sub-blocks with mycelium on exposed surface (80 g each), samples ‘A’, ‘B’, ‘C’, ‘D’, see Fig. 2a.
- 1 collection of fruiting bodies (50 g), sample ‘E’, see Fig. 2b.
- 4 sub-blocks with substrate exposed surface (80 g each), samples ‘F’, ‘G’, ‘H’, ‘I’, see Fig. 2c.
- 1 collection of substrate fragmented into loose seeds (80 g), sample ‘J’

Each portion was put in a 2 L, plastic container with a removable airtight lid (model 1720ZS KLIP IT, Sistema, NZ). The divider in the middle allows air flow between the two sides. On the other side, two 50 g sachets of dry silica gel (model WD-1, Viola Technology Ltd, UK) that could hold more than 30 g of water together were put, as shown in the bottom half of Fig. 2. The weight of silica gel was recorded daily for 41 days.

### Electrical activity mapped to depth

**Fig. 3:**
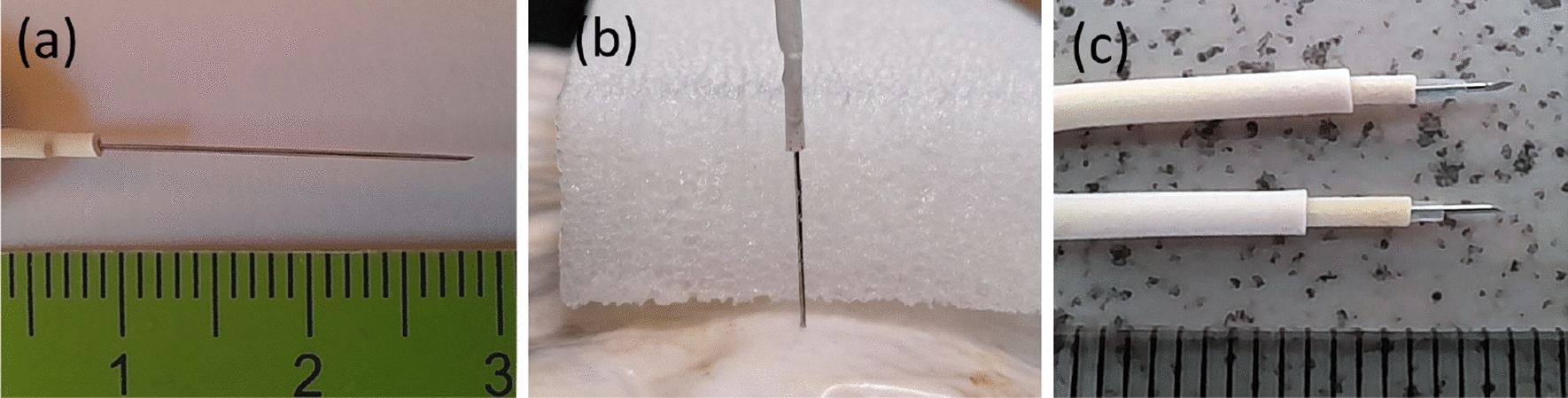
Three electrode configurations (**a**) unmodified sub-dermal needle inserted directly (**b**) unmodified sub-dermal needle inserted through foam spacer (**c**) partly insulated sub-dermal needles so only tips exposed

Three configurations of electrodes were used to record electrical activity at different depths in mycelium-bound composite:

- unmodified sub-dermal needle electrodes (18 mm length) inserted $\sim$ 15 mm depth into body of spawn substrate, see Fig. 3a.
- unmodified sub-dermal needle electrodes inserted through 20 mm thick foam spacer so inserted $\sim$ 3 mm depth into mycelium, see Fig. 3b.
- sub-dermal needle electrodes partly electrically insulated (with 16 mm of heat shrink tubing) inserted $\sim$ 18 mm into body of spawn substrate to make electrical contact $\sim$ 16 mm to 18 mm below surface, see Fig. 3c.

### Electrical response to water droplets on mycelium surface

**Fig. 4:**
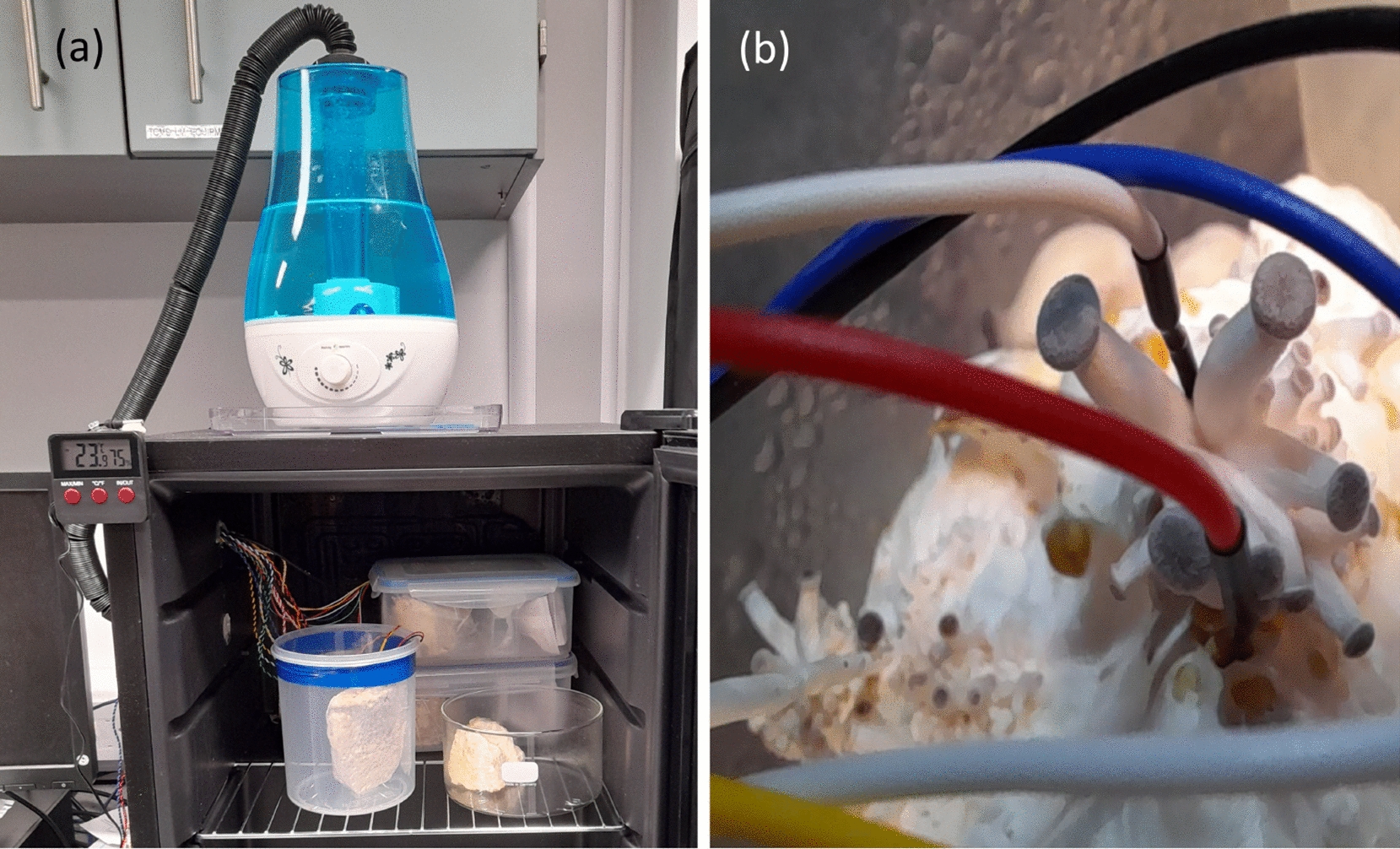
**a** Bespoke environmental chamber. **b** Electrodes inserted into fungi

Samples of spawn substrate blocks (with a myceliated surface) were taken out of their dehydration containers and the electrical activity was measured with stainless steel sub-dermal needle electrodes, see Fig. 4b, and a data logger (ADC-24, PICO Technology Ltd, UK). For experimental flexibility and consistency, recordings were made inside a custom-made environmental chamber, as shown in Fig. 4a. Temperature control (e.g. 10 ± 1 $^\circ$C) was done with a digital thermostat that controlled how the cooling compressor worked. The temperature and humidity inside the chamber were measured with a digital thermo-hygrometer (76114, Trixie Ltd, UK). The humidity in the environmental chamber was raised to $\sim$ 75 % using an ultrasonic humidifier (3 L Silent, Hffheer Ltd) filled with deionised water and activated for 15 min every 3 h, see Fig. 4a.

In a separate experiment, a fresh block of substrate (1 L bag) that had been well colonised with *Pleurotus ostreatus* was taken out of the bag and left to dry in the air. To record electrical activity, (18 mm length) needle electrodes were inserted $\sim$ 15 mm below the skin. After 24 h, the surface of the mycelium was sprayed by hand with de-ionised water. The results are shown in "Results" shown in Fig. 13.

**Fig. 5:**
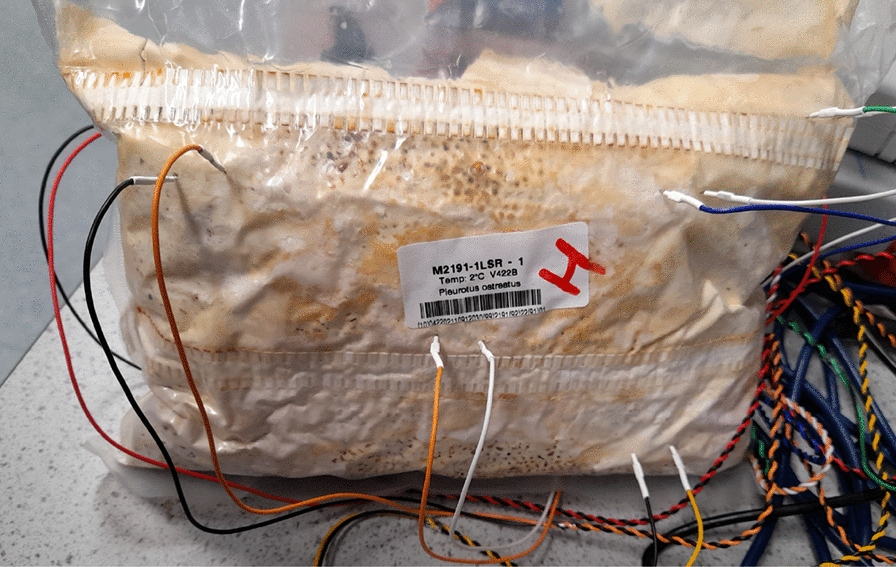
Water droplets manually sprayed onto mycelium surface inside open plastic bag with electrodes inserted from outside

In a different experiment, to make sure that water on the surface of the mycelium didn’t change the electricity conductivity between the electrodes, a block of substrate that had been well colonised with *Pleurotus ostreatus* was left inside a plastic bag with only the top part of the bag open so it could slowly dry out. Eight pairs of subdermal needle electrodes (18 mm length) were inserted (15 mm) through the sides of the plastic bag into the body of the spawn substrate (see Fig. 5, Spray top bag). After spontaneous spike trains ceased, de-ionized water was sprayed by hand onto the surface of the mycelium through the open end of the bag, keeping the electrodes away from the sprayed water. Results shown in Fig. 14.

## Results

### Moisture content mapped to electrical conductivity

Three blocks of well colonised substrate (two species) were allow to dry under ambient conditions (18 to 22 °C) for 10 d to 12 d, see Table  1.

**Table 1:** Moisture content of fresh blocks of spawn substrate (from commercial suppliers)

Species	Weight block (g)	Drying period (day)	Moisture content at start (%)	Moisture content at end (%)	Moisture content change (%)
*Hericium erinaceus*	∼ 750	12	∼ 99	∼ 9	∼ 90
*Pleurotus ostreatus* ‘A’	∼ 500	11	∼ 92	∼ 18	$\sim$ 74
*Pleurotus ostreatus* ‘B’	∼ 500	10	∼ 82	∼ 7	∼ 75

**Fig. 6:**
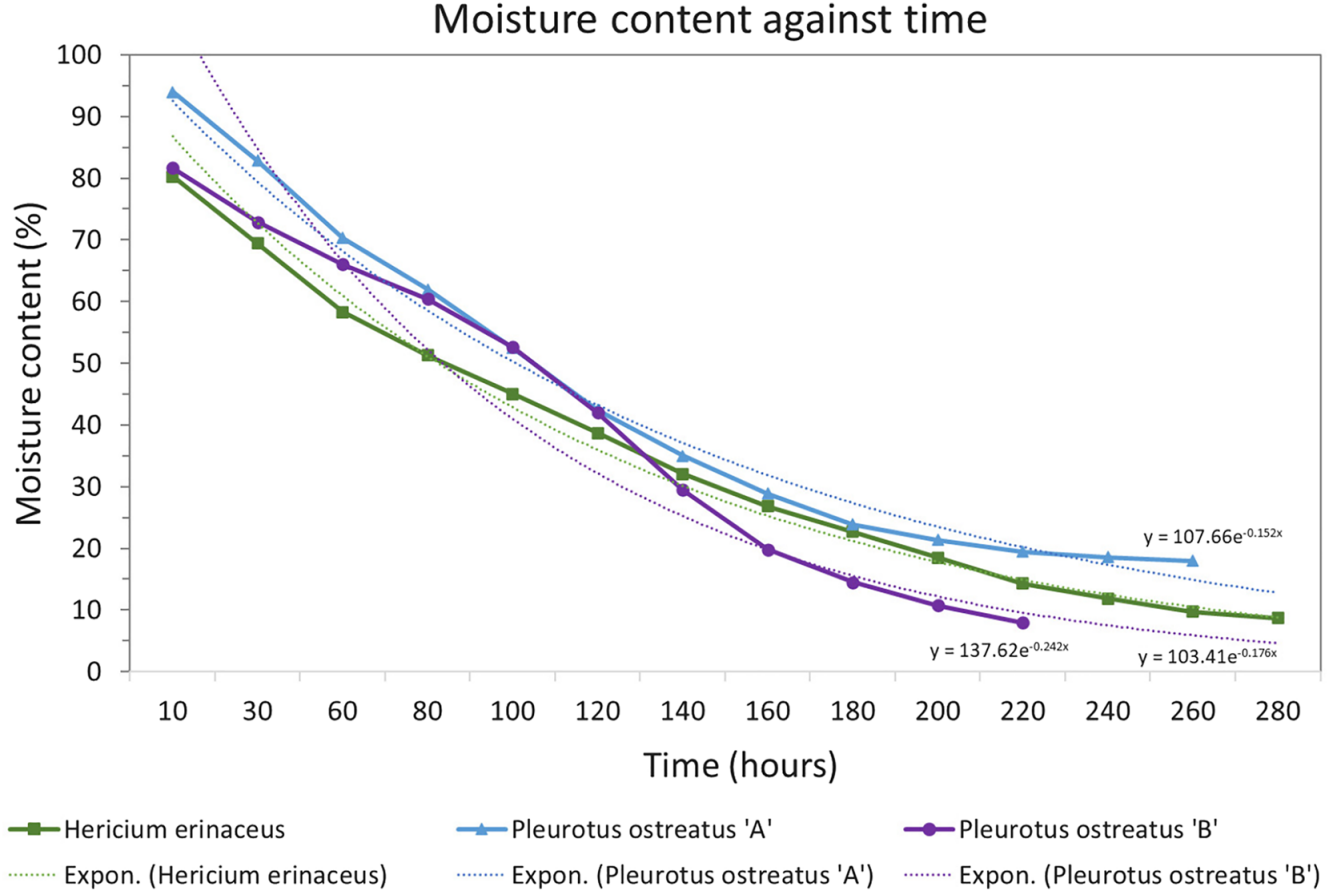
Rates of dehydration of *Hericium erinaceus* and *Pleurotus ostreatus* blocks

Rate of dehydration inferred from HOBO electrical conductivity probe for *Hericium erinaceus* and *Pleurotus ostreatus* are shown in see Fig. 6.

### Electrical activity mapped to moisture content

**Fig. 7:**
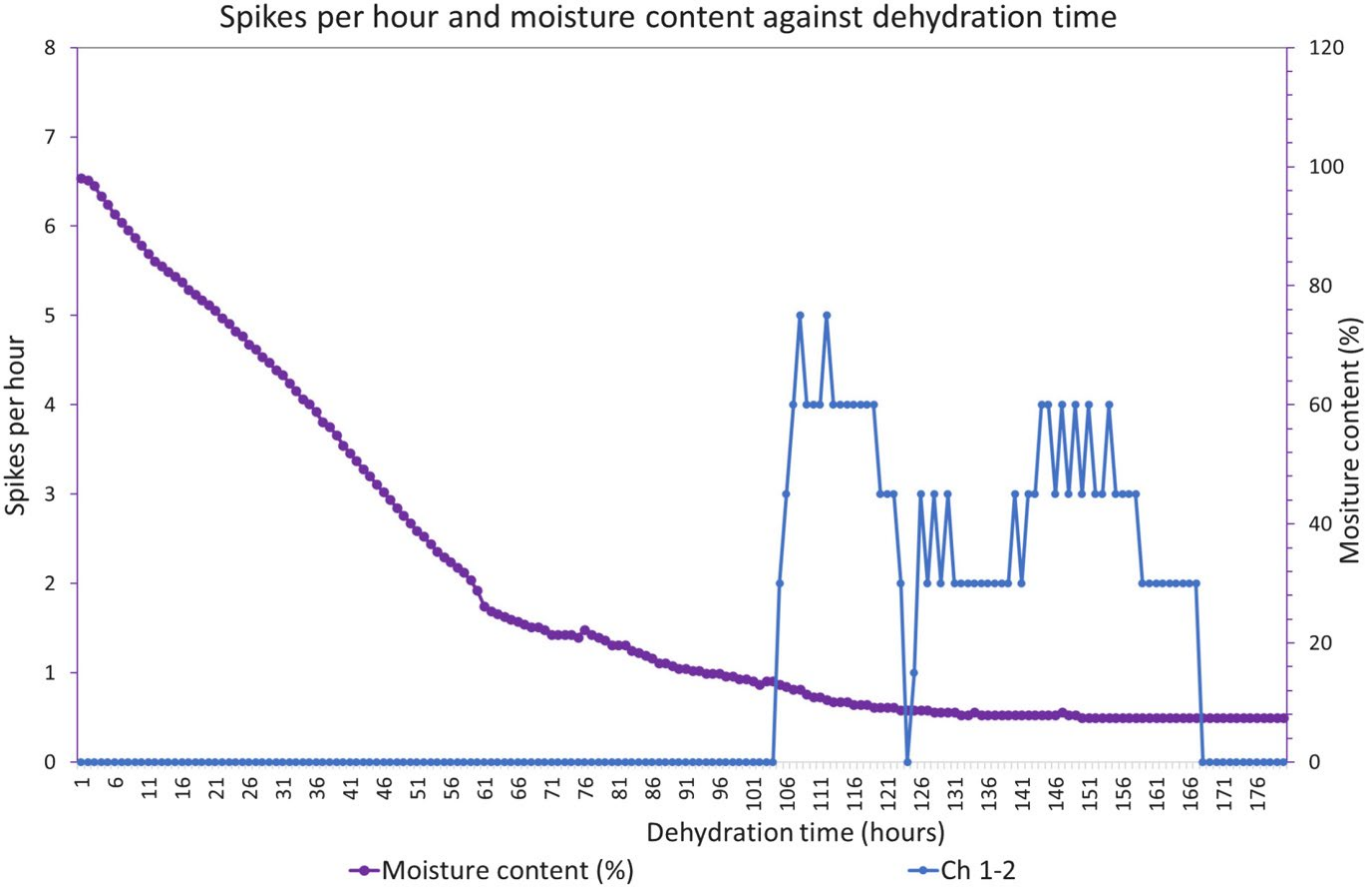
Electrical activity against moisture content over time of unwrapped block of substrate colonised with *Pleurotus ostreatus*

An exemplar of electrical activity against moisture content in unwrapped block of substrate colonised with *Pleurotus ostreatus* is shown in Fig. 7. In this example, spike trains spontaneously initiated after 106 h and ceased after 168 h.

**Fig. 8:**
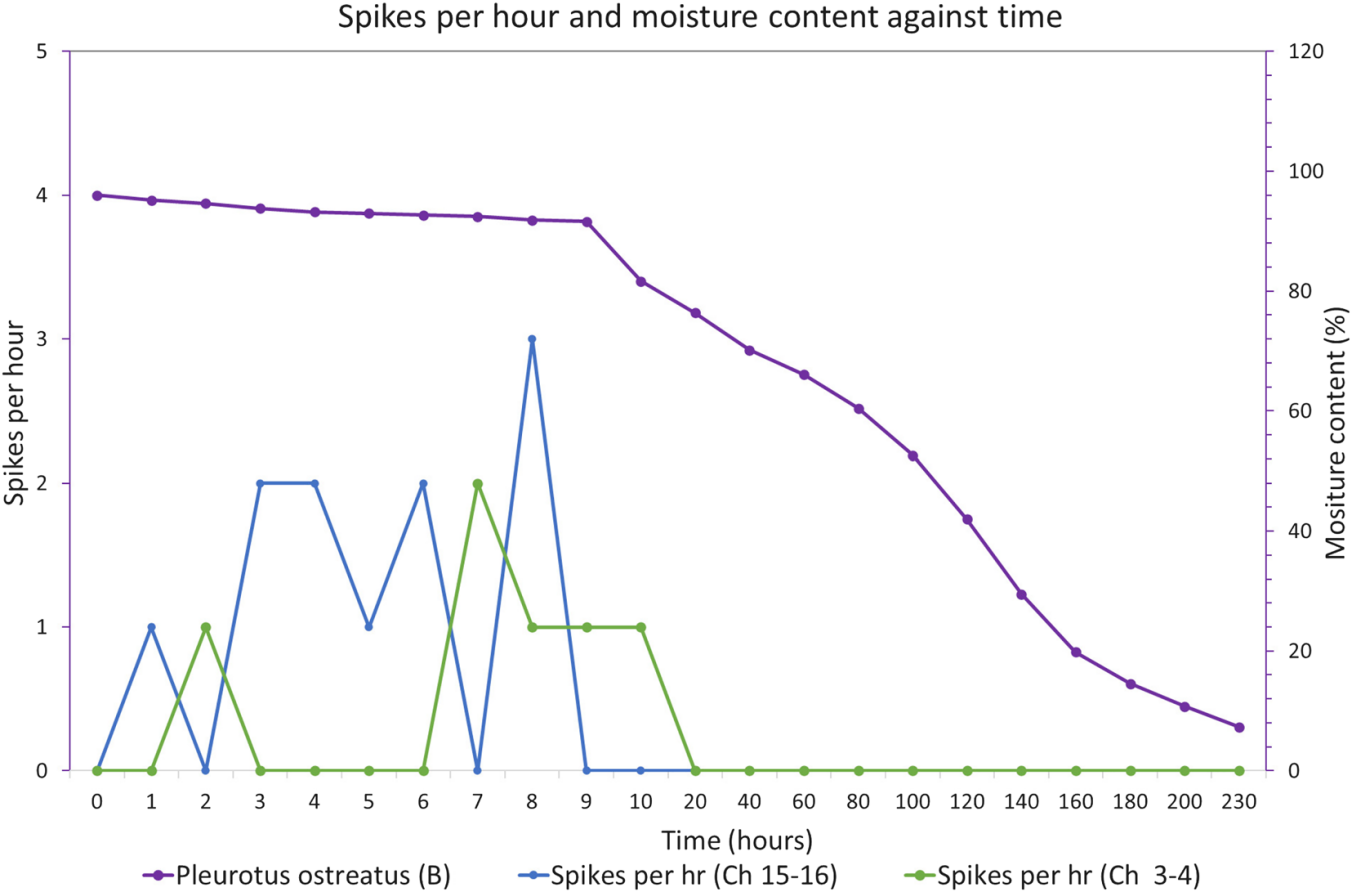
Electrical activity against moisture content over time in a partly wrapped block of substrate colonised with *Pleurotus ostreatus*

An exemplar of electrical activity against moisture content in partly wrapped block of substrate colonised with *Pleurotus ostreatus* is shown in Fig. 8. In this example, spontaneously spike trains are recorded from the start and ceased after $\sim$ 20 h at which time the moisture content has dropped to $<\sim$ 70%.

### Rate of water loss from substrate colonised with fungi

**Fig. 9:**
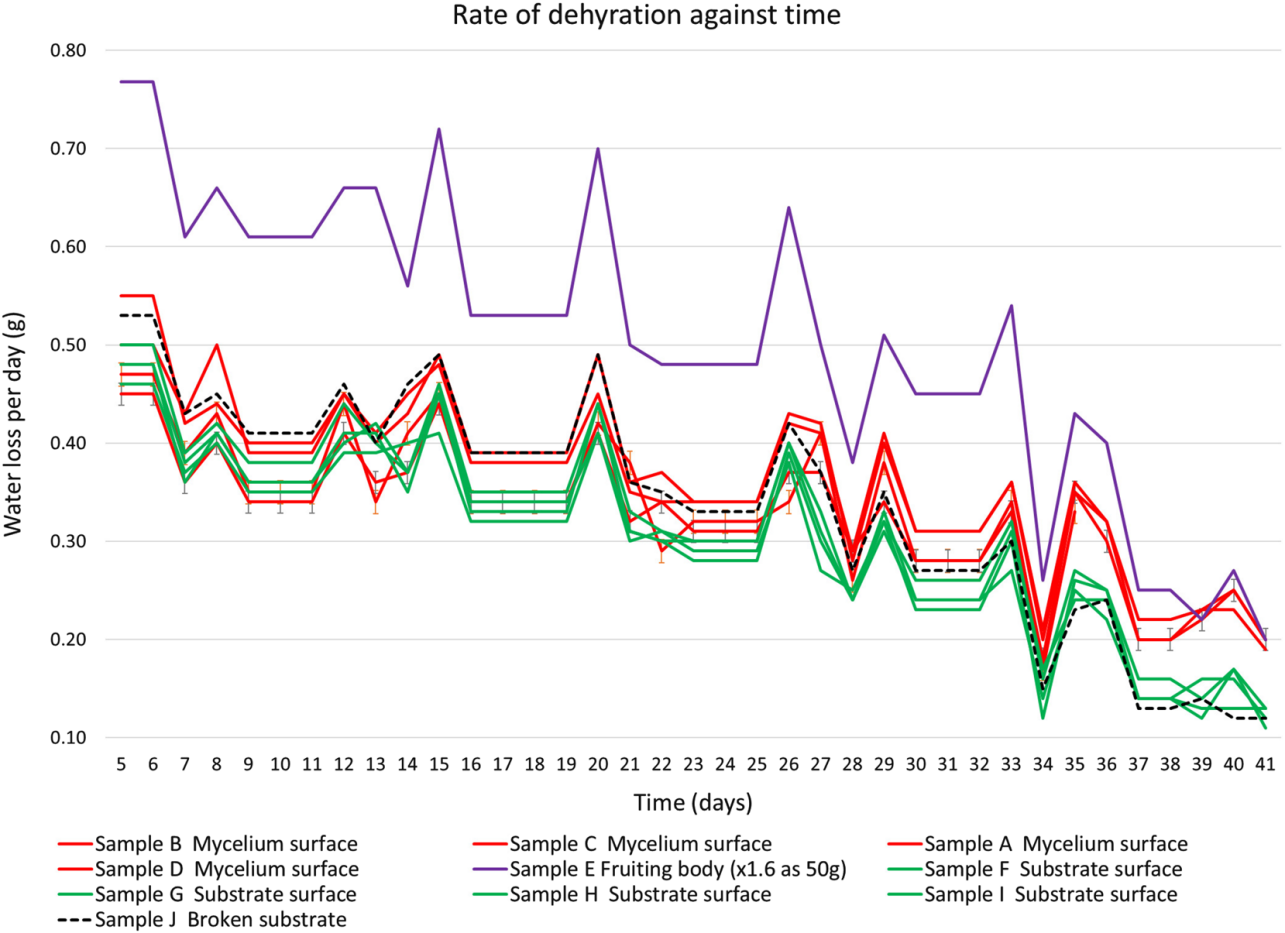
Rate of water loss from colonised substrate and fruiting bodies against time

The rate of water loss from substrate colonised with fungi and fruiting bodies in low humidity air against time is shown in Fig. 9. To simplify the comparison, the rate of water loss from fruiting bodies was adjusted pro rota ($\times$ 1.6) to compensate for a smaller sample mass (50 g rather than 80 g).

### Electrical activity mapped to depth

**Fig. 10:**
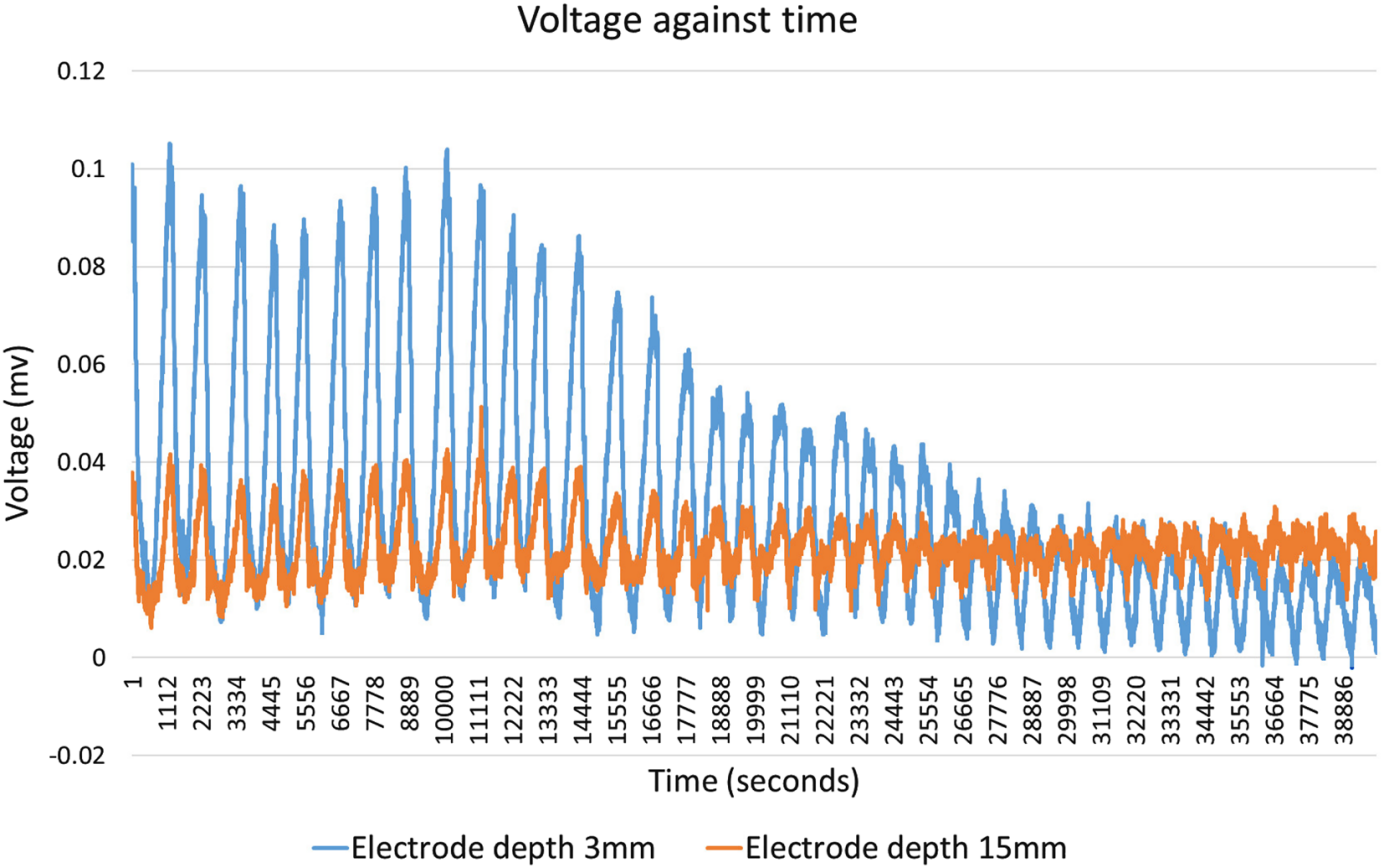
Exemplar of electrical activity recorded with electrodes inserted 3 mm and 15 mm

A comparison of the magnitudes of electrical potentials of trains of electrical spikes recorded with exposed electrodes inserted 3 mm and 15 mm into the same block of substrate colonised with *Pleurotus ostreatus* is shown in Fig. 10.

**Fig. 11:**
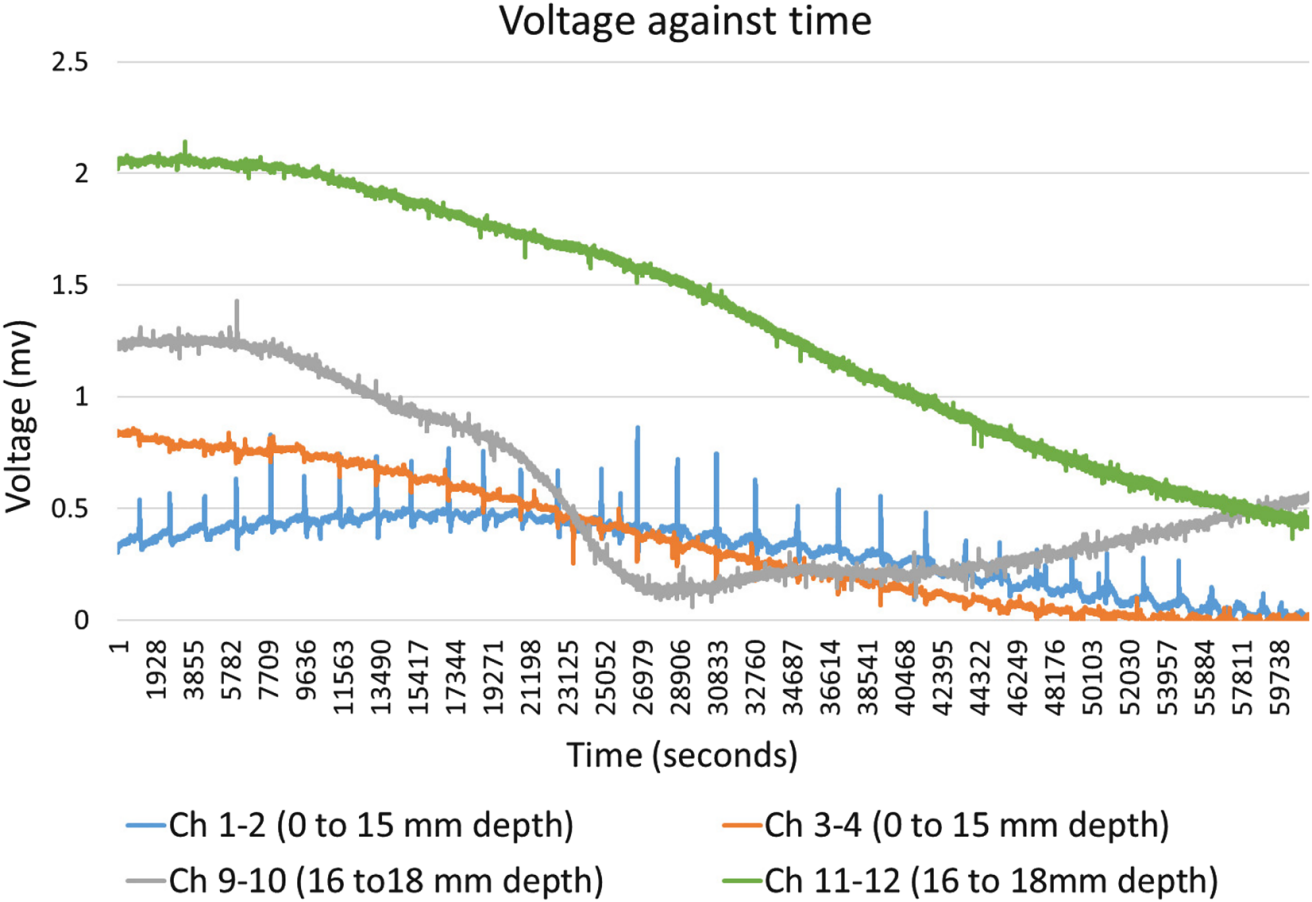
Exemplar of electrical activity recorded with exposed electrodes inserted 15 mm and 16 mm to 18 mm

Figure 11 shows Ch1–2 and Ch3–4 with exposed electrodes inserted 15 mm and Ch9–10 and Ch11–12 with exposed electrodes 16 mm to 18 mm into the same block of substrate colonised with *Pleurotus ostreatus*.

### Electrical response to water droplets on mycelium surface

**Fig. 12:**
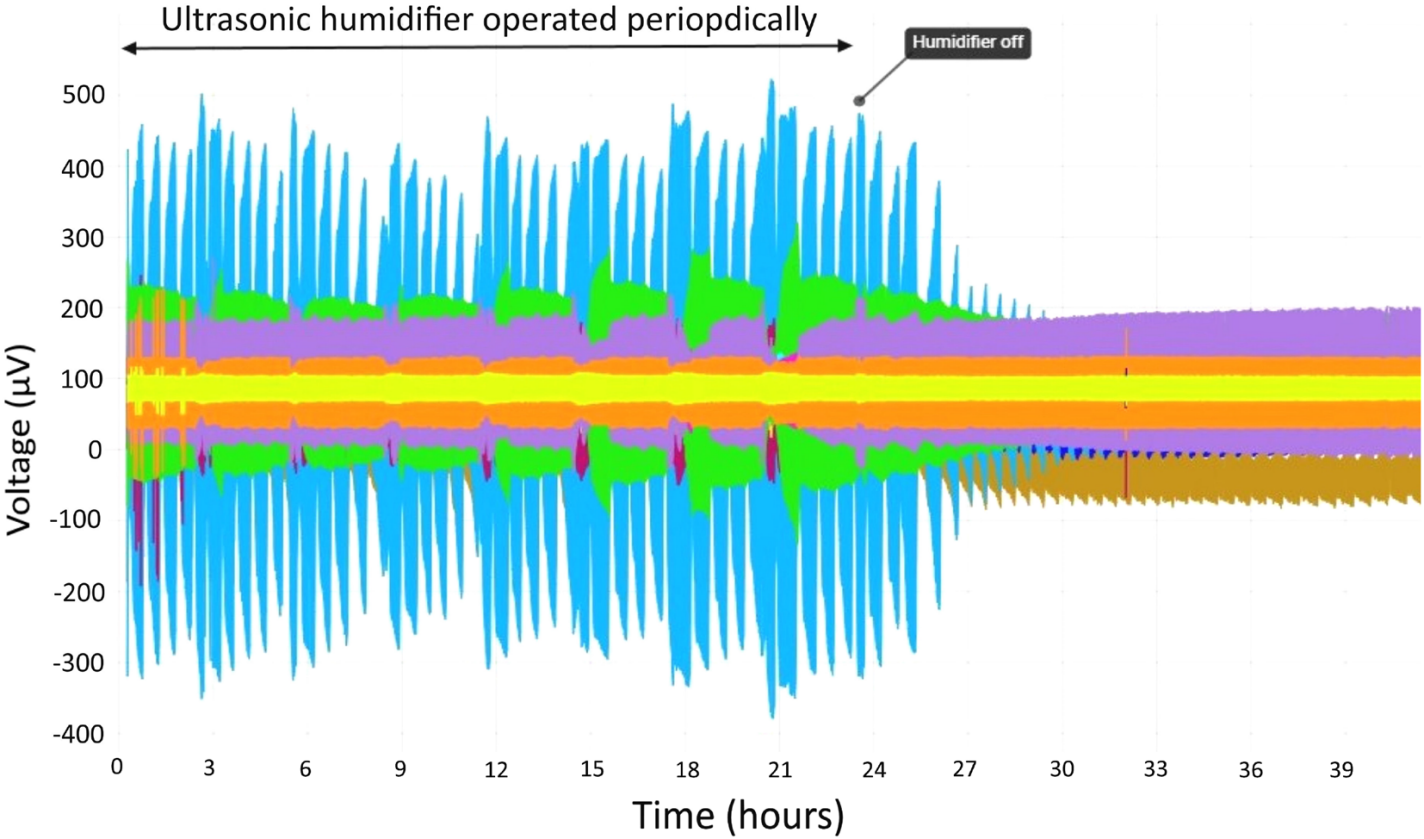
Exemplar of electrical activity during and after periodic operation of humidifier

Multiple trains of electrical spikes were recorded during and immediately after operation of the ultrasonic humidifier, see Fig. 12.

**Fig. 13:**
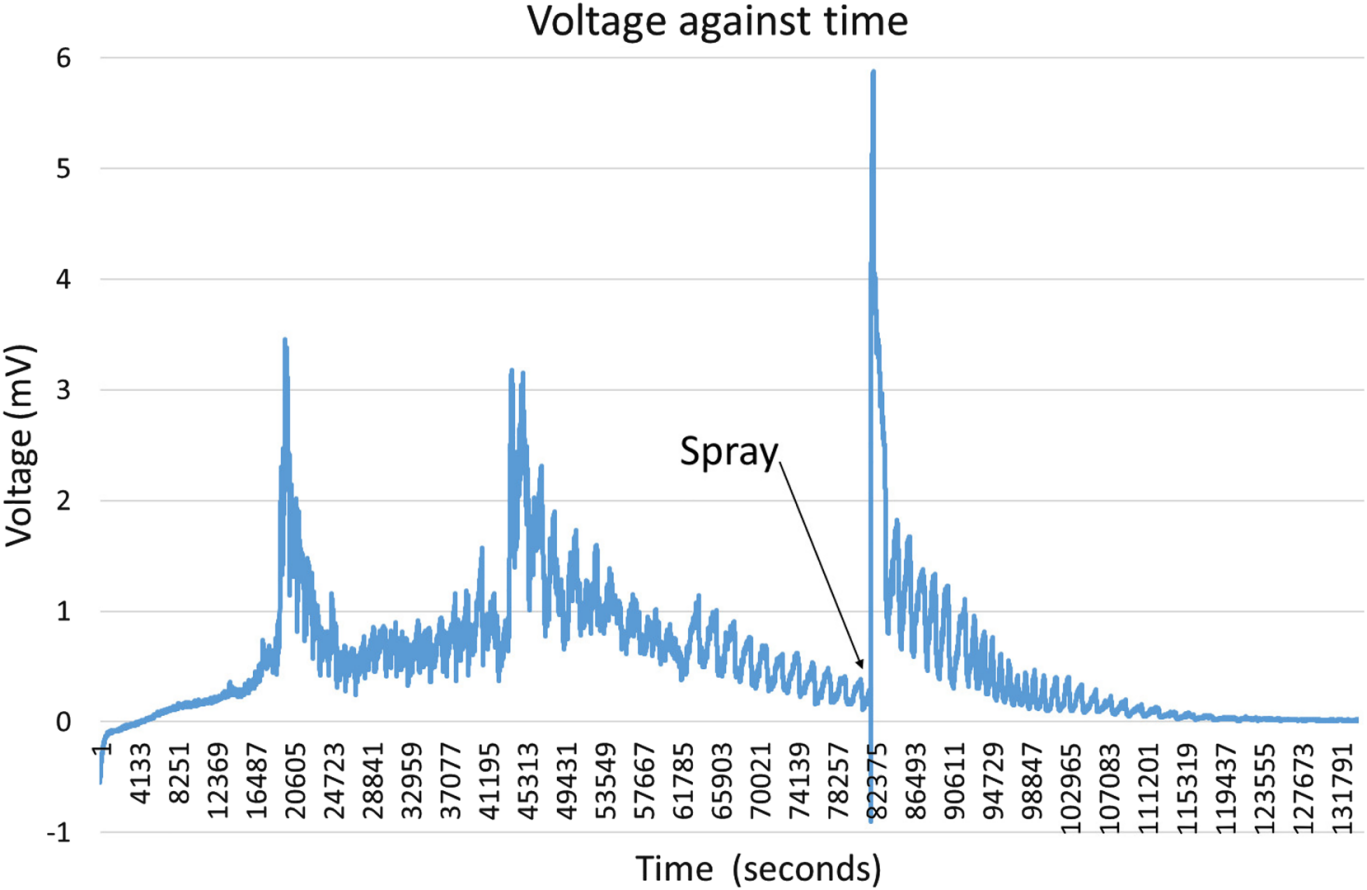
Exemplar of spike trains triggered both spontaneously and from water droplets sprayed onto the myceliated surface

Spike trains triggered both spontaneously and from water droplets sprayed onto the surface of the fresh block of spawn substrate are shown are Fig. 13.

**Fig. 14:**
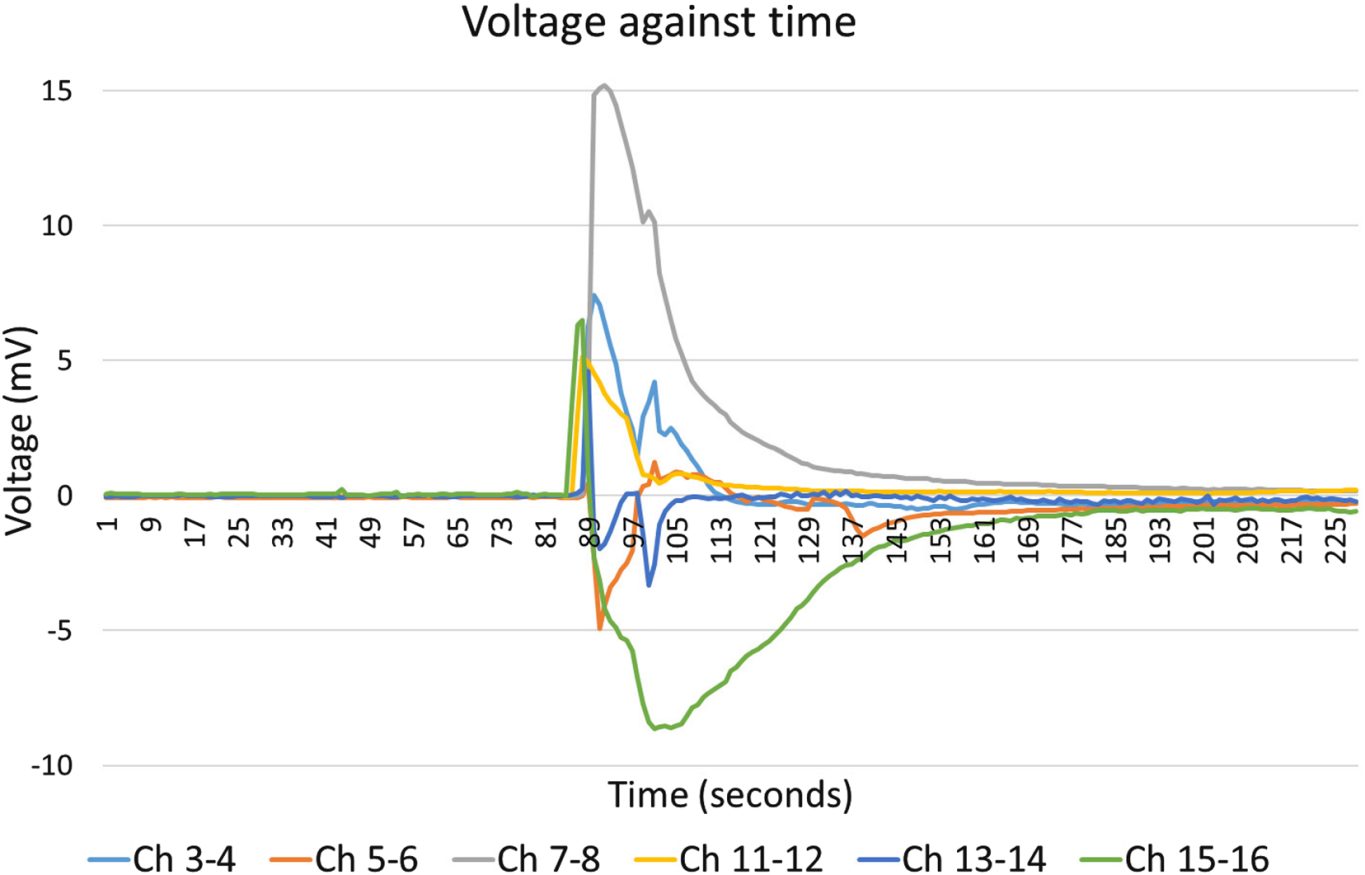
Exemplar of electrical activity before, during and after manually spraying water droplets onto the surface of mycelium inside the bag (after spontaneous spike trains ceased)

After spontaneous spike trains ceased, electrical activity before, during and after manually spraying water droplets onto the myceliated surface inside the bag is shown in Fig. 14.

## Discussion

Obtaining fresh blocks ($\sim$ 500 g) of electrically active spawn substrate from commercial suppliers at desired times was challenging (e.g. limited stock availability, some blocks didn’t show electrical activity). Further, most commercial suppliers were unwilling to provide details of substrate composition (beyond “rye or millet seeds” as considered a ‘trade secret’). Therefore, variation in substrate might exist between both batches from the same supplier and different suppliers. Further, the level of colonisation of blocks varied greatly between suppliers and times of recordings as the fungi consumed the substrate as a source of nutrients. Additionally, the heterogeneous mixture of substrate and fungi added an additional variable.

### Moisture content mapped to electrical conductivity

Despite an extensive search, no commercial available moisture probe calibrated for substrate colonised with fungi was found. Therefore, it was necessary to calibrate a general-purpose moisture probe for this purpose. Moisture probe HOBO EC-5 and H21 Micro Station from Tempcon Instrumentation Ltd were selected and readings were calibrated by weighting samples before and after oven drying to determine water content. The large physical size of the EC-5 probes meant blocks of spawn substrate needed to be sufficiently large (e.g. 500 g) to avoid them splitting when the EC-5 probes were inserted into them (which would have interfered with electrical conductivity measurements).

An electrical path from the EC-5 probes via HOBO H21 Micro Station to the data logging computer appeared to interfere with the recording of electrical potentials made with PICO ADC-24. Therefore, it was necessary to unplug the USB connection between HOBO H21 Micro Station and computer during recordings (utilising Micro Station internally batteries for power).

The initial moisture content of fresh spawn substrate blocks (as supplied) is high (typically 80% to 100%) over time (several days) in low humidity air ($\sim$ 30% relative humidity) the moisture content reduces significantly (< 20 %). The drying curves of colonised substrate were found to be similar to organic material (such as seeds and vegetables) dehydrating in air [38–41].

### Electrical activity mapped to moisture content

Electrical spikes were not initially recorded in unwrapped fresh blocks of colonised substrate. For example, see Fig. 7. However, as the moisture content dropped (e.g. <20 %) the fungi become stressed and spikes spontaneously initiated (e.g. 3 ± 3h$^{-1}$). After a further period of dehydration, the rate spikes decreased and finally ceased (e.g. $\sim$ 64 h).

Partly wrapped fresh blocks of colonised substrate typically exhibit markedly different electrical characteristics. For example, Fig. 8 shows a partly wrapped block of substrate colonised with *Pleurotus ostreatus* dehydrating. In this example, spontaneously spike trains are recorded from the start and ceased after $\sim$ 20 h at which time the moisture content has dropped to $<\sim$ 70%.

The rates of dehydration shown in Figs. 7 and 8 against time are different as the latter is partly enclosed in an (open) plastic bag which slows the rate of water loss from the block.

### Rate of water loss from substrate colonised with fungi

The rate of water loss from fruiting bodies was considerably higher (40–60%) than substrate both with and without mycelium skin. This suggests that linear cytoplasmic units [42, 43] are drawing water near the surface which increases the rate of evaporation (in an environment with low-humidity air). The rate of water loss from the substrate with a mycelium skin became increasingly higher (10–60%) than substrate without mycelium skin.

Initially, the rate of water loss from the fragmented substrate (mostly loose rye and millet grain seeds) was slightly higher than the average substrate both with and without mycelium skin. However, over time this situation reversed and the rate of water loss become slightly lower than the average substrate both with and without mycelium skin. This suggests that the greater surface area of the fragmented substrate becomes less important as the substrate’s moisture content is reduced close to bone dry.

After $\sim$ 35 d the measured rate of ‘water loss’ levelled off which suggests the remaining measurement of weight gain was water absorbed from the ambient air when the plastic enclosures were opened to remove the sachets of silica gel for weighing. Measuring the weight of the silica gel (rather than directly weighting the sample) provides several advantages including no loss of material or infection of the sample during the weighting process.

### Electrical activity mapped to depth

Recordings of electrical activity measured just below the surface (0 mm to 3 mm) typically contain higher potential differences than those recorded across a broader depth (0 mm to 15 mm) inside the block, as shown in Fig. 10. This difference is also noticeable in recordings electrodes inserted 0 mm 15 mm compared to 16 mm to 18 mm. For example, Ch 1–2 and 3–4 compared to Ch 9–10 and 11–12 in Fig. 11. This suggests that electrical potential is discharged by electrodes making electrical connections to others parts of the block which are less electrically active. In other words, the high electrical conductivity of the metal electrodes discharges voltage differential as current through other low conductivity parts of the block.

Inserting sub-dermal needles at a significant depth (e.g. 15 mm) into the colonised substrate provides mechanical support to hold the electrodes in position. If sub-dermal needles are only inserted a shallow depth (e.g. 3 mm) then any movement of the flying leads can disturb the connection between the electrode and mycelium. To overcome this issue, a foam spacer was first glued to the surface of the plastic bag containing the spawn substrate. Unmodified sub-dermal needles were then inserted through 20 mm thick foam spacer to securely positioned them $\sim$ 3 mm depth into mycelium. Other methods of securing the electrodes’ positions (e.g. holding the top of the electrodes with a frame rigid relative to the block, electrically insulating the bottom part of the needle and inserting further into the substrate, etc) are possible.

Electrical potential was also observed to vary with electrode separation. A distance of $\sim$ 20 mm between centres of electrodes was found to be effective for monitoring electrical activity, as was evidenced by the identification of significantly more and larger spikes in the recordings. This suggests that there is an optimum spacing for the electrodes in any environment.

Optimising the relative physical positions of electrodes in colonised substrate (in terms of both depth and spacing) is important to maximising the sensitivity of monitoring and interconnections to other systems.

### Electrical response to water droplets on myceliated surface

Figure 12 shows water droplets condensing on the surface of mycelium from the high humidity air (in this example from the ultrasonic humidifier) can trigger trains of electrical spikes. Spikes of diminishing electrical potential continue to occur for $\sim$ 2 h after the humidifier is switched off.

In fresh spawn substrate, spikes can initiate both spontaneously and/or be triggered by water droplets on the surface in mycelium. For example, in Fig. 13, trains of spikes ($\sim$ 3 mV peak) trigger spontaneously $\sim$ 10 h period. Spraying with de-ionised water triggers a spike train of twice the voltage potential ($\sim$ 6 mV peak). Electrical potential in fruiting bodies with precipitation has been reported [44]. The influence of environmental conditions on the electrical activity of fruiting bodies has been reported [45].

If the spawn substrate is allowed to partly dehydrate, spontaneous spike trains cease. However, electrical response (non-spike train) to water droplets still occurs. Exemplar Fig. 14 shows significant electrical pulses ($\sim$ 15 mV peak), most pulses contain two peaks, the initial one is larger followed ($\sim$ 15s later) by a second peak. If the spawn substrate is allowed to completely dehydrate, no electrical response to water droplets on the surface of mycelium occurs.

## Conclusions

Electrical activity in fresh well colonised substrate is significantly greater if part or all of the surface is enclosed with an impermeable layer (e.g. flexible plastic bag or rigid plastic container). For example, trains of electrical spikes initiate spontaneously in fresh spawn substrate blocks with $>\sim$ 65% moisture content inside an (open) plastic bag. If the substrate is unwrapped and allowed to partly dehydrate spikes can spontaneously occur at $<\sim$ 15% moisture content. The rate of change of moisture content in the substrate is affected by how well the substrate’s surface is colonised with fungi. In particular, the higher the proportion of the surface covered with fungi the quicker the substrate dehydrates. In fresh spawn, electrical spikes can initiate both spontaneously and/or be triggered by water droplets on the surface in the mycelium. If the spawn is allowed to partly dehydrate, spontaneous spike trains cease. However, electrical response (non-spike train) to water droplets can still occur. The versatility of fungi, in terms of being able to tailor different biofabricated configurations such as composites, flexible tissue, rhizomorphs, and foamy materials, provides a promising opportunity for the development of unconventional computing systems. The suitability of utilising fungi for particular applications needs to be carefully assessed (e.g. quantitative analysis of the selected species) as some fungi form mycotoxins or might become invasive species [46].

## Data Availability

The raw datasets obtained in this study are available from the corresponding author on reasonable request.
